# Several circulating miRNAs related to hyperlipidemia and atherosclerotic cardiovascular diseases

**DOI:** 10.1186/s12944-019-1046-z

**Published:** 2019-04-22

**Authors:** Jiang Xu, Zixuan Chen, Yingge Wang, Xiaohong Wang, Lu Chen, Tingting Yuan, Xiangming Tang, Yaoyao Lu, Hongmei Chen, Miaolei Chen, Zuowei Duan, Jianglin Fan, Jingyan Liang, Xinjiang Zhang

**Affiliations:** 1 Department of Neurology, Affiliated Hospital of Yangzhou University, Yangzhou, 225009 China; 2 Institute of Translational Medicine, Medical College, Yangzhou University, Yangzhou, 225009 China; 3 Jiangsu Key Laboratory of Integrated Traditional Chinese and Western Medicine for Prevention and Treatment of Senile Diseases, Yangzhou University, Yangzhou, 225001 China; 4 Jiangsu Co-Innovation Center for Prevention and Control of Important Animal Infectious Disease and Zoonoses, Yangzhou, 225009 China; 5 Department of Molecular Pathology, Faculty of Medicine, Graduate School of Medical Sciences, University of Yamanashi, Yamanashi, 409-3898 Japan

## Abstract

**Background:**

In recent years, an increasing number of studies have proved that circulating miRNAs could be used for the early diagnosis of cardiovascular diseases and even play vital roles in the evaluation of therapeutic effects or prognosis. This study was conducted to examine the correlation between serum microRNAs and hyperlipidemia to provide a theoretical basis for the early screening and intervention of atherosclerotic cardiovascular diseases (ASCVD).

**Methods:**

The serum samples and clinical data of 122 patients with hyperlipidemia and 168 healthy subjects were collected. Related clinical information was statistically analyzed for the two groups. Expression of circulating miRNAs was detected by miRNA microarray analysis and further verified by reverse transcription-quantitative PCR (RT-qPCR).

**Results:**

Statistical analysis of clinical information revealed a significant difference in the incidence of ASCVD between the two groups. The MiRNA microarray analysis (*n* = 10) showed 22 miRNAs with significantly different expression, among which 12 showed upregulation, and the others showed downregulation. Those possessing obvious differences and stable expression in the miRNA microarray, including miRNA-191-3p, miRNA-933, and miRNA-425-3p, were chosen for further investigation using RT-qPCR. The results demonstrated that several miRNAs were related to lipid metabolism disorders, especially miRNA-933. The area under the curve (AUC) of miRNA-933 in distinguishing the hyperlipidemia and ASCVD patients was 0.739 (95% CI, 0.682–0.795; *P* < 0.01) and 0.703 (95% CI, 0.643–0.763, *P* < 0.01), respectively.

**Conclusions:**

In conclusion, miRNA-191-3p, miRNA-933, and miRNA-425-3p may be depressed in the peripheral circulation of patients with lipid metabolism disorders (mainly LDL). Circulating miRNA-933 could be a feasible predictor for ASCVD at the early stage.

**Electronic supplementary material:**

The online version of this article (10.1186/s12944-019-1046-z) contains supplementary material, which is available to authorized users.

## Introduction

Atherosclerotic cardiovascular disease (ASCVD) is defined as a group of clinical diseases mostly caused by endothelial dysfunction based on atherosclerosis (AS), including coronary heart disease, stroke, and other various cardiovascular diseases [1]. These diseases seriously threaten human life and result in high mortality and disability rates all over the world. The pathogenesis of ASCVD is extremely complex, mainly including lipid infiltration, endothelial damage reactions, and chronic inflammatory damage. The dysregulation of lipid metabolism, particularly the low-density lipoprotein (LDL), is commonly considered the foundational cause resulting in AS [1, 2]. Numerous studies have shown that LDL could enter in the intima or middle membrane through the damaged vascular endothelium, eventually forming oxidized LDL (ox-LDL), which was widely believed to be the fundamental pathological agent of atheromatous plaques [3]. A variety of factors influence the formation of atherosclerosis, including hypertension, hyperlipidemia, hyperglycemia, obesity, smoking, etc. These risk factors comprise the majority of the key targets for prevention or treatment of ASCVD, among which lipid metabolism disorders, especially the abnormal elevation of circulating LDL, is the most important point. Presently, controlling circulating LDL levels has become the most important measure to prevent ASCVD [4, 5].

MiRNAs are a class of endogenous, highly conserved, non-coding RNAs, mostly consisting of 19–24 nucleotides. MiRNAs could regulate gene expression on the posttranscriptional level by binding to the 3’UTR of mRNA, which may either inhibit the translation of protein from mRNA or promote the degradation of mRNA. These RNAs participate in nearly all physiological and pathological processes in organisms [6]. For ASCVD, various miRNAs have been reported that could affect lipid metabolism and the formation of atherosclerosis through regulating the function of vascular smooth muscle cells, endothelial cells, macrophages, and so on [7–9]. Numerous studies have proved that miRNAs could exist stably in peripheral blood circulation and have shown good physiological characteristics to tolerate different temperatures, pH, storage times, and even repeated freezing and thawing. Additional studies revealed the dynamic alterations of circulating miRNA expression could be detected under different pathological conditions, including various cardiovascular diseases, thereby indicating that detecting the different expression of miRNAs in peripheral blood circulation may help in the early diagnosis of atherosclerotic cardiovascular diseases [10, 11].

In fact, an increasing number of studies have shown that noncoding ribonucleic acids could be used for the early diagnosis of cardiovascular diseases and even play vital roles in the evaluation of therapeutic effects or prognosis [12, 13]. Wang GK et al. demonstrated that plasma miR-208a might be a novel biomarker for acute myocardial infarction [14]. Fang Y et al. found that miR-10a could regulate pro-inflammatory phenotypes in atherosclerosis-susceptible endothelium [15]. The exact involvement of circulating miRNAs in cardiovascular diseases remains to be clarified. The aim of this study was to compare circulating miRNA profiles in hyperlipidemic patients characterized by hyper-LDL-C in comparison to normolipidemic controls to provide assistance for early screening of ASCVD.

## Materials and methods

### Study subjects

Subjects, including healthy volunteers, outpatients, and in-patients, were recruited from October 2017 to December 2018 at Yangzhou University, Affiliated Hospital of China. According to the blood lipid level (mainly the serum LDL-C level) of patients, the subjects were divided into a hyperlipidemia group and a healthy control group.

The inclusion criteria for patients with hyper-LDL-C was according to the classification criteria of dyslipidemia in the population in 2016 Chinese guideline for the management of dyslipidemia in adults, defined hyper-LDL-C as serum LDL-C level ≥ 3.4 mmol/L(130 mg/dl). As there are small number of patients with isolated hyper-LDL-C in clinical, some with mixed dyslipidemia were also recruited. The exclusion criteria of the study was as follows: age > 80 years, autoimmune diseases, systemic inflammatory diseases, cancer, rheumatic heart diseases, thyroid disease, or liver and kidney dysfunction. Patients with a history of major trauma and surgery, or women who were pregnant and breastfeeding were also not included in this study. Clinical information, including personal history, heart disease, cerebrovascular disease, and genetic history information, etc., was collected from all patients. Hypertension was diagnosed as systolic pressure 140 mmHg and/or diastolic blood pressure 90 mmHg. The diagnostic criteria of diabetes were fasting plasma glucose 7.0 mmol/L or random glucose 11.1 mmol/L. The diagnosis of ASCVD was made if the patient had a history of one or more of the following: acute coronary syndromes, myocardial infarction, stable or unstable angina, coronary or other arterial revascularization, stroke, and TIA. Similar principles were used to collect other medical history information. Subjects who continuously or cumulatively smoked for more than 6 months were defined as smokers. Alcoholics were defined as individuals with an average daily consumption of alcohol equivalent to that of pure alcohol (125 g), which lasted for more than 10 years. The study was approved by the ethics committee of The First People’s Hospital of Yangzhou. All the patients were given informed consent and recruited into the ongoing prospective study.

### Blood sample collection and RNA extraction

In a calm environment, fasting venous blood was collected in a test tube without anticoagulant. After centrifugation, the serum was removed and stored at − 80 °C. The miRNA expression profiles of 10 serum samples (*n* = 5 for each group) were detected by miRNA microarray (Agilent miRNA chip V2.4, Agilent Technologies, Santa Clara, CA, USA). Serum miRNA was isolated using a miRcute serum miRNA kit (Tiangen Biotech, Beijing, China), according to the manufacturer’s instructions. Total RNA was quantified by the NanoDrop ND-2000 (Thermo Scientific) and the RNA integrity was assessed using Agilent Bioanalyzer 2100 (Agilent Technologies). The sample labeling, microarray hybridization and washing were performed based on the manufacturer’s standard protocols. Briefly, total RNA was dephosphorylated, denaturated and then labeled with Cyanine-3-CTP. After purification the labeled RNAs were hybridized onto the microarray. After washing, the arrays were scanned with the Agilent Scanner G2505C (Agilent Technologies). Differentially expressed miRNAs were then identified through fold change (FC) as well as *P* value calculated using t-test. The threshold set for up- and down-regulated genes was a fold change≥2.0 and a *P* value≤0.05.

For RT-qPCR experiments, the purity and concentration of miRNA extracted from the serum was determined with a spectrophotometer. Only RNA samples with an A260/A280 ratio of 1.9–2.1 were used for reverse transcription of cDNA.

### RT-qPCR determination

The miRcute miRNA cDNA First-Strand Synthesis kit (Tiangen Biotech) was used for reverse transcription of miRNA extracted, according to the manufacturer’s instructions. The reaction system (20 μL) contained the following: 10 μL 2 x miRNA RT reaction buffer, 2 μL miRNA RT enzyme mix, and 8 μL total RNA. The reaction conditions were as follows: 25 °C for 5 min, 42 °C for 60 min, and 95 °C for 3 min. After cDNA synthesis, DEPC water was used to dilute the product in a 1:5 ratio, and the samples were stored at − 20 °C. A miRcute-enhanced miRNA Fluorescence Quantitative Detection kit (SYBR Green), miRNA primers (miRNA-320b, miRNA-933, miRNA-191-3p, and U6), and miRcute miRNA fluorescence quantitative detection reagents were used for detection, with a fluorescence quantitative PCR (Roche, Basel, Switzerland) instrument. The 20-μL reaction system was prepared according to the manufacturer’s instructions as follows: 2 μL template cDNA (5 times dilution), 10 μL 2x miRcute miRNA premix, 0.4 μL miRNA/U6 primers, 0.4 μL reverse primer, and 7.2 μL RNase-free water. The reaction conditions were as follows: 95 °C for 15 min; 94 °C for 20 s, 64 °C for 30 s, and 72 °C for 34 s for 5 cycles, no fluorescence signal; 94 °C for 20 s; and 64 °C for 30 s for a total of 40 cycles. All samples were examined with at least two duplicates. We used U6 as an internal reference in the qPCR experiment, the relative amount of miRNA normalized to U6 was calculated with the Equation 2^-ΔΔCT^, in which ΔΔCT = (CT _miRNA_ − CT_U6_) _target_ − (CT _miRNA_ − CT _U6_) _control_.

### Statistical methods

The experimental data was analyzed with SPSS13.0 software (SPSS, Inc., Chicago, IL, USA). The mean ± standard deviation (x ± s) was used as the quantitative index, and differences between the two groups were compared by *t* tests. The count data were compared with the χ^2^ test. Receiver operating characteristic curves were also used to analyze relevant experimental data. *P* < 0.05 was considered statistically significant.

## Results

We first collected and analyzed the clinical information of the patients between the hyperlipidemia and control groups. Table 1 shows the basic clinical data of the subjects of the two groups. According to the table, there were no significant differences in age, smoking history, drinking history, and incidence of diabetes between the two groups. Similarly, differences in the history of stroke, hypertension, and body mass index were also not obvious. Nevertheless, our dates revealed a significant difference in the incidence of both carotid atherosclerosis and ASCVD between the two groups. In terms of biochemical indicators, uric acid showed a significant difference between the two groups (Table 1).

**Table 1:** Basic clinical data of subjects in the LDL group and control group

Terms	Hyperlipidemia group (*n* = 122)	Normal group (*n* = 168)	*P*-value
Age (years)	50.06 ± 1.09	48.601 ± 0.84	0.407
Sex (male/female)	74/48	69/99	0.378
History of smoking (Smoker/Nonsmoker)	45/77	60/108	0.522
History of alcoholism (Yes/No) NoNONo)Nondrinker)	42/80	64/104	0.520
Body mass index (BMI)	24.56 ± 0.22	23.72 ± 0.24	0.064
Hypertension (Yes/No)	38/84	68/100	0.103
Diabetes (Yes/ No)	26/96	50/118	0.106
Carotid plaque (Yes/ No)	46/56	47/99	0.039
Not checked	20	22	
History of stroke (Yes/ No)	25/97	22/146	0.092
History of coronary heart disease	33/89	34/134	0.174
ASCVD	53/69	41/119	0.002
Fatty liver	41/81	34/134	0.101
Random blood glucose	5.81 ± 0.27	5.33 ± 0.06	0.126
Creatinine	70.6 ± 1.36	71.78 ± 3.17	0.349
Urea nitrogen	5.33 ± 0.13	5.60 ± 0.13	0.083
Uric acid	345.68 ± 7.50	321.4 ± 6.41	0.013
AST	20.4 ± 0.78	19.9 ± 0.42	0.507
ALT	24.93 ± 1.45	21.73 ± 1.12	0.111
TC	6.34 ± 0.14	4.37 ± 0.28	0.024
TG	1.77 ± 1.07	2.01 ± 0.59	0.067
HDL-C	1.16 ± 0.53	1.58 ± 0.31	0.021

**Fig. 1:**
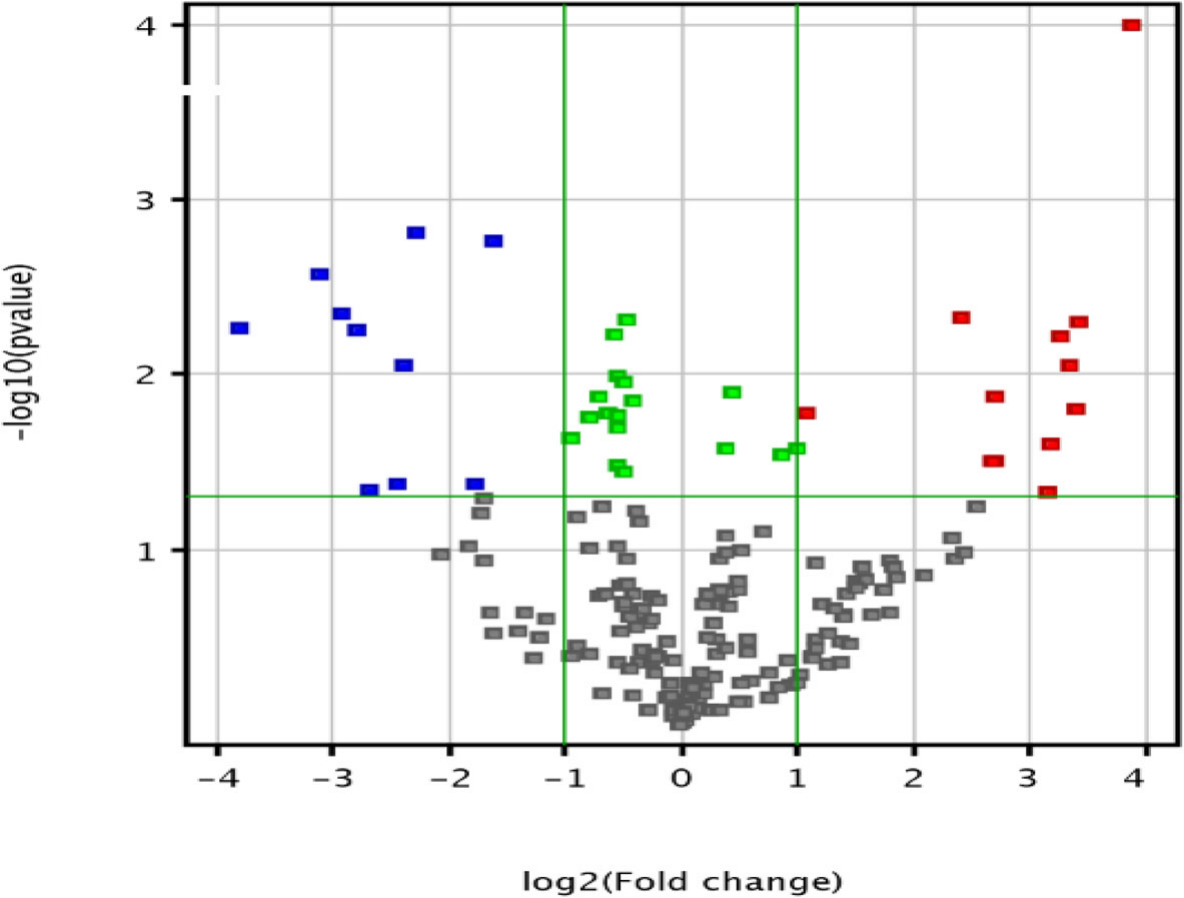
Volcano plot showing miRNAs detected by microarray. The red dots represent differential upregulation of miRNAs (fold change> 2,*P* > 0.05). The blue dots represent differential downregulation of miRNAs (fold change<−2,*P* < 0.05). The green points represent miRNAs (absolute fold change < 2,*P* < 0.05). The gray dots indicate the miRNAs expressed with *P*-value>0.05

**Fig. 2:**
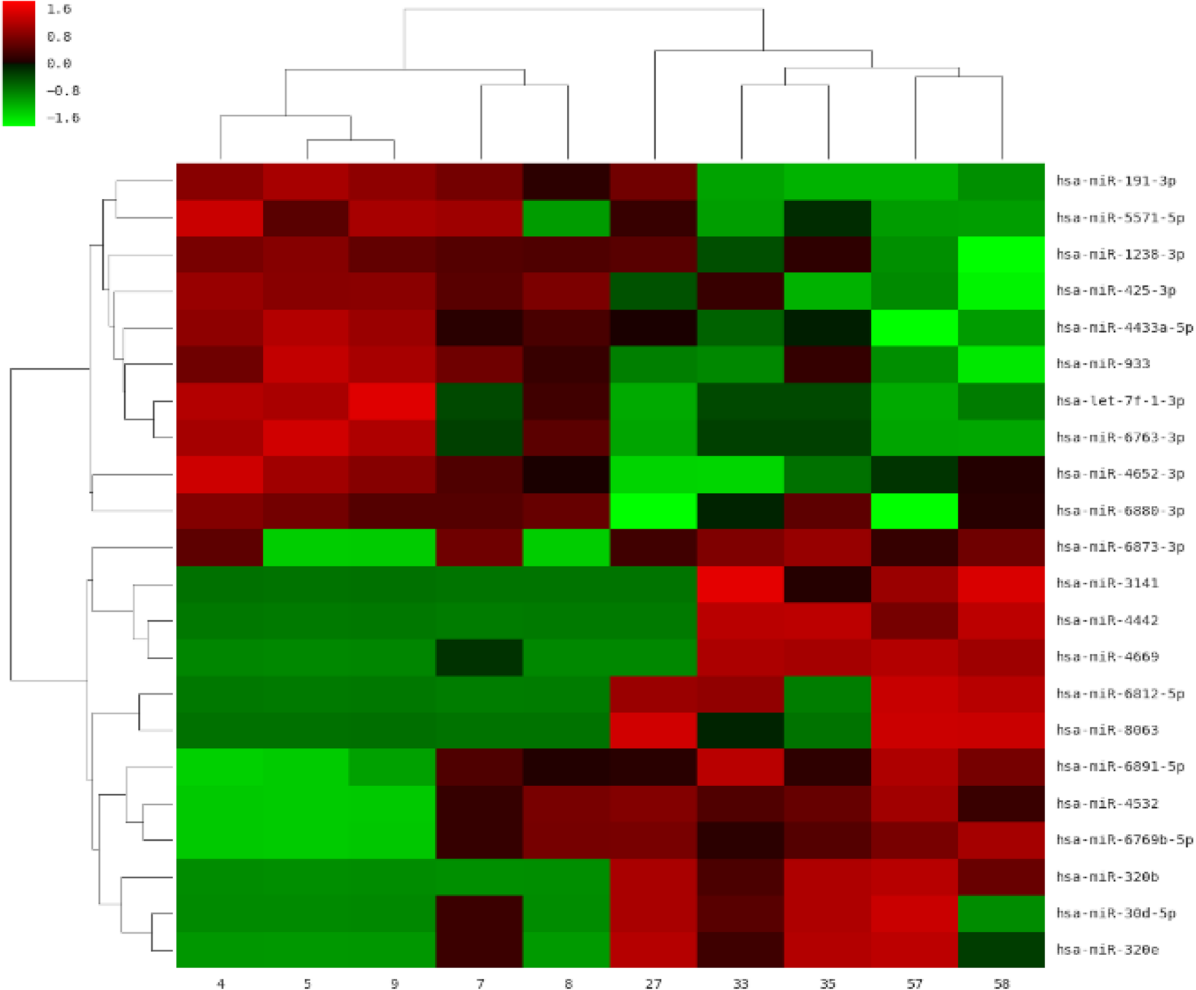
Heat map of microarray results. The rows indicate miRNAs and columns indicate samples. The red color represents upregulation, and the green color represents downregulation. Samples 4, 5, 7, 8, and 9 were from the normal group, and samples 27, 33, 35, 57, and 58 were from the hyperlipidemia group

We then randomly selected 10 serum samples from subjects of the two groups, with 5 patients from the hyperlipidemia group and 5 patients from the control group, for miRNA microarray analysis. A total of 2449 miRNAs with intensity > 30 in at least one test serum were detected, as shown in the volcano plot (Fig. 1). Compared with the control group, 28 differentially expressed miRNAs were identified in the hyperlipidemia group, including 12 upregulated miRNAs and 10 downregulated miRNAs, at the criteria of FC (Fold change) ≥2 and *P* ≤ 0.05 (Fig. 2). Detailed differential expression of miRNAs is shown in Table 2.

**Table 2:** Fold-change and *P*-values of the 28 differentially expressed miRNAs

Systematic_name	*P*-value	Fold-change	Regulation
hsa-miR-320b	5.64E-06	14.652229	up
hsa-miR-425-3p	0.001549529	4.8689585	down
hsa-miR-933	0.0017513	3.0571334	down
hsa-miR-6763-3p	0.002649574	8.668792	down
hsa-let-7f-1-3p	0.004478273	7.5040197	down
hsa-miR-6812-5p	0.004728028	5.341505	up
hsa-miR-4442	0.00502631	10.697879	up
hsa-miR-191-3p	0.005391372	13.9024725	down
hsa-miR-4652-3p	0.005592787	6.952554	down
hsa-miR-320e	0.006005395	9.612401	up
hsa-miR-4433a-5p	0.008774483	5.2011013	down
hsa-miR-4669	0.008905013	10.235651	up
hsa-miR-3141	0.01339152	6.5122175	up
hsa-miR-8063	0.015623125	10.531111	up
hsa-miR-6891-5p	0.01648203	2.1228352	up
hsa-miR-30d-5p	0.025248433	9.162879	up
hsa-miR-6769b-5p	0.031363655	6.3925323	up
hsa-miR-4532	0.03136994	6.5774345	up
hsa-miR-1238-3p	0.042109374	3.418031	down
hsa-miR-6880-3p	0.042388383	5.4697547	down
hsa-miR-5571-5p	0.04509465	6.394996	down
hsa-miR-6873-3p	0.04718673	8.873202	up

**Fig. 3:**
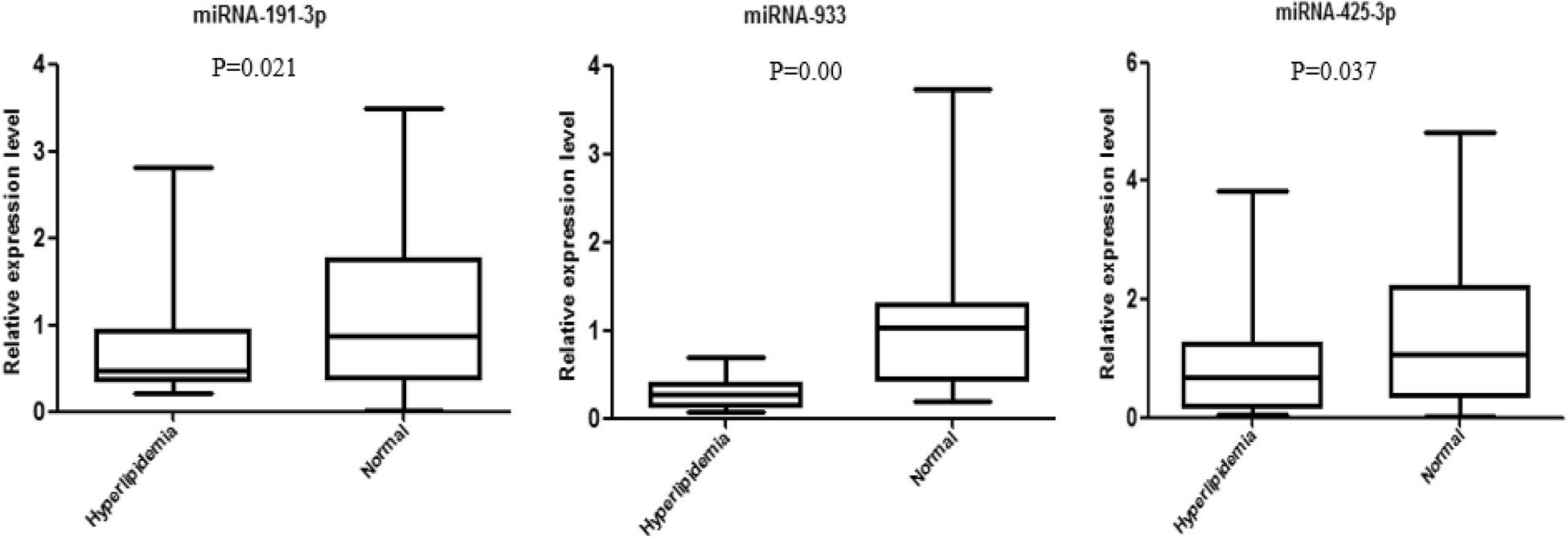
Expression levels of miRNA-191-3p, miRNA-933, and miRNA-425-3p were consistently downregulated in patients with hyperlipidemia compared with healthy volunteers, thereby supporting the microarray results. All of the expression levels of these three miRNAs were normalized to U6. (*n* = 20)

According to the *P* value and the FC value, three miRNAs with stable expression (miRNA-191-3p, miRNA-933, and miRNA-425-3p) as well as significant differences between the two groups were selected for further RT-qPCR validation. We randomly chose 40 other serum samples with 20 from the hyperlipidemia group and 20 from the control group for preliminary PCR validation. The results showed that the expression of all three miRNAs were downregulated, which is consistent with the results of the miRNA microarray (Fig. 3).

**Fig. 4:**
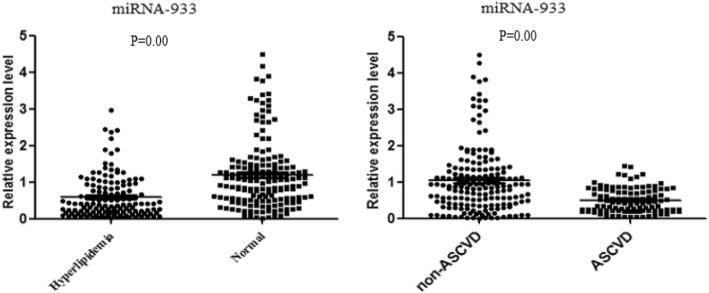
miRNA-933 was significantly downregulated in patients with hyperlipidemia (*n* = 122) compared with healthy volunteers (*n* = 168) (Left); The expression of miRNA-933 in ASCVD group (*n* = 96) was also significantly lower than that in non-ASCVD group (*n* = 194) (Right). Expression levels are normalized to U6. *P* < 0.05 was considered statistically significant

Based on the result of the preliminary PCR experiment, we selected miRNA-933 for further validation via additional experiments to detect the expression levels of miRNA-933 in serum samples from a larger sample with 290 subjects. As Fig. 4 shows, miRNA-933 was significantly downregulated in the hyperlipidemia group in comparison with the healthy volunteers (*P* = 0.00). Pearson correlation coefficient was also conducted to analyzed the correlation between LDL-C and miRNA-933 in the hyperlipidemia group, the result showed LDL-C and miRNA-933 were moderately correlated (R = − 0.691) (Additional file 1: Figure S1). Considering the results described above, we have sufficient reason to believe that circulating miRNA-933 is related to the disorder of serum lipid metabolism, especially hyper-LDL-C .

**Fig. 5:**
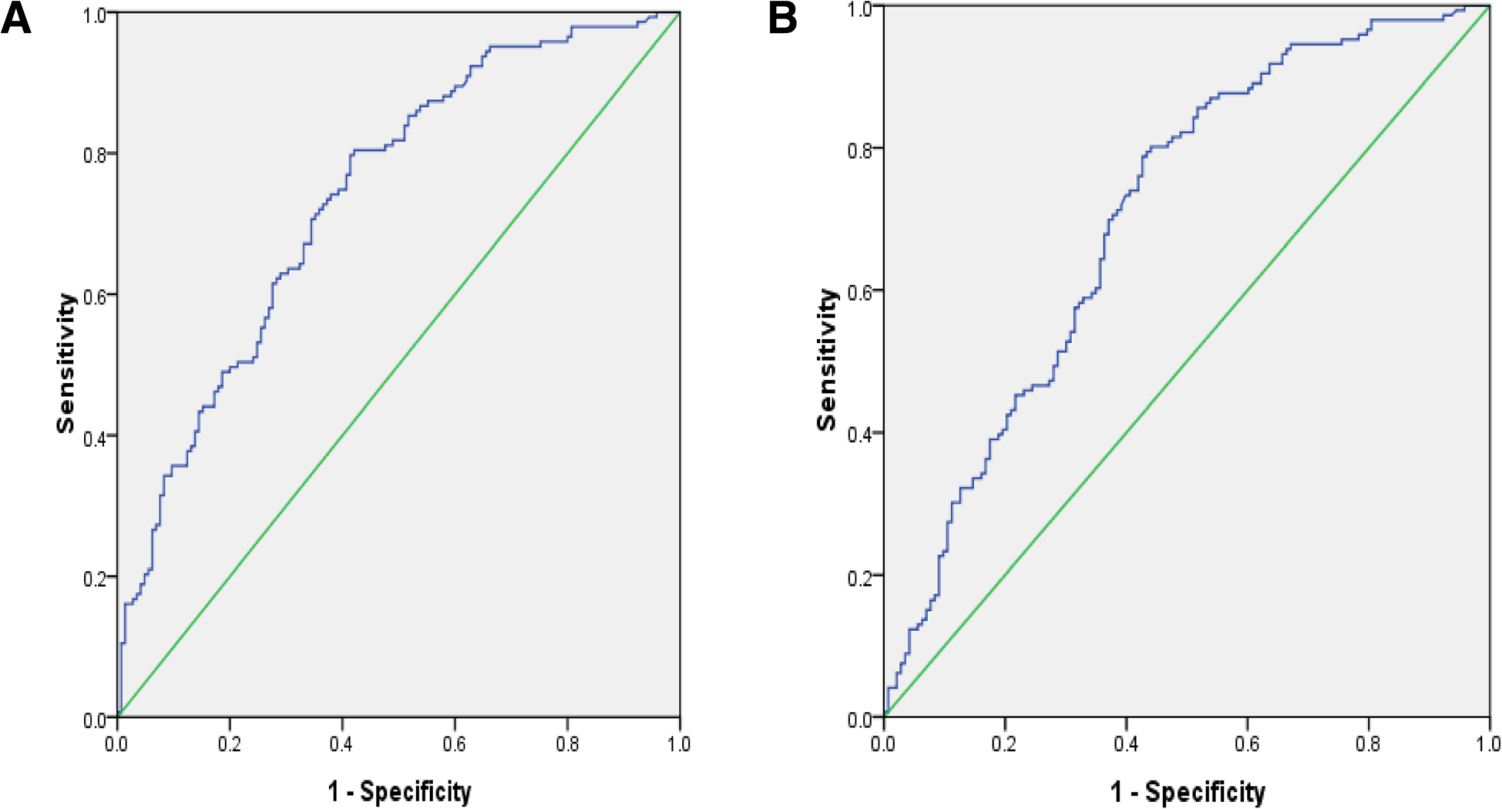
Graphs showing receiver operating characteristic curve analyses of serum miRNA-933 levels. **a** In the analysis for distinguishing hyperlipidemia patients from healthy volunteers, the area under the curve (AUC) was 0.739 (95% CI, 0.682–0.795; *P* = 0.00); **b** In distinguishing the ASCVD patients, the AUC was 0.703 (95% CI, 0.643–0.763; *P* = 0.00). *P* < 0.05 was considered statistically significant

Since miRNA-933 is probably related to the disorder of serum lipid metabolism, Based on quartiles, the expression of miRNA-933 in ASCVD and non-ASCVD was further compared. The result showed that the expression of miRNA-933 in ASCVD group was significantly lower than that in non-ASCVD group (*P* = 0.00) (Fig. 4). After adjusting the variables *P* < 0.1 in univariate analysis (e.g. smoker (*p* = 0.079), LDL-C (*P* = 0.012), TC (*P* = 0.031), HDL (*P* = 0.026), and TG (*P* = 0.046)). Logistic regression analysis showed that the first quartile of serum miRNA-933 (OR 10.701, 95% CI, 2.834–40.48, *P* = 0.000), the second quartile (OR 5.255, 95% CI, 0.875–12.111; *P* = 0.018) were independent risk factors for ASCVD (Additional file 1: Table S1).

we continuously performed ROC curve analyses to investigate the usefulness of miRNA-933 as a diagnostic marker for ASCVD. The area under the curve (AUC) was 0.739 (95% CI, 0.682–0.795; *P* = 0.00) in distinguishing the hyperlipidemia patients (Fig. 5a), as well as 0.703 (95% CI, 0.643–0.763; *P* = 0.00) in distinguishing the ASCVD patients (Fig. 5b).

## Discussion

ASCVD, known as the main reason of death among elderly people, generally includes the following: acute coronary syndromes, history of myocardial infarction, stable or unstable angina, coronary or other arterial revascularization, stroke, TIA, or peripheral arterial disease presumed to be of atherosclerotic origin, according to the 2013 ACC/AHA guidelines [1]. The dysregulation of lipid metabolism, especially the abnormal elevation of LDL-C, is considered to be the main pathological basis of ASCVD [16]. At present, the circulating level of LDL-C is the most extensive diagnostic indicator for assessing and predicting the risk of atherosclerosis, and it has been globally acknowledged that reducing circulating LDL-C levels will effectively help in preventing ASCVD [14, 17]. Therefore, early intervention to inhibit circulating LDL-C levels has causally become more and more important.

In this study, we first statistically analyzed the clinical data of patients with abnormal elevation of LDL-C and normal controls. The results showed that the rates of ASCVD and carotid atherosclerosis were significantly higher in the hyperlipidemia group compared with the healthy control (*P*-values = 0.002, 0.039, respectively), thereby demonstrating the high correlation between circulating LDL-C with ASCVD. Our data also showed there were no significant differences in age, smoking history, body mass index, cerebrovascular disease, hypertension, diabetes, coronary heart disease, or other factors between the two groups.

**Fig. 6:**
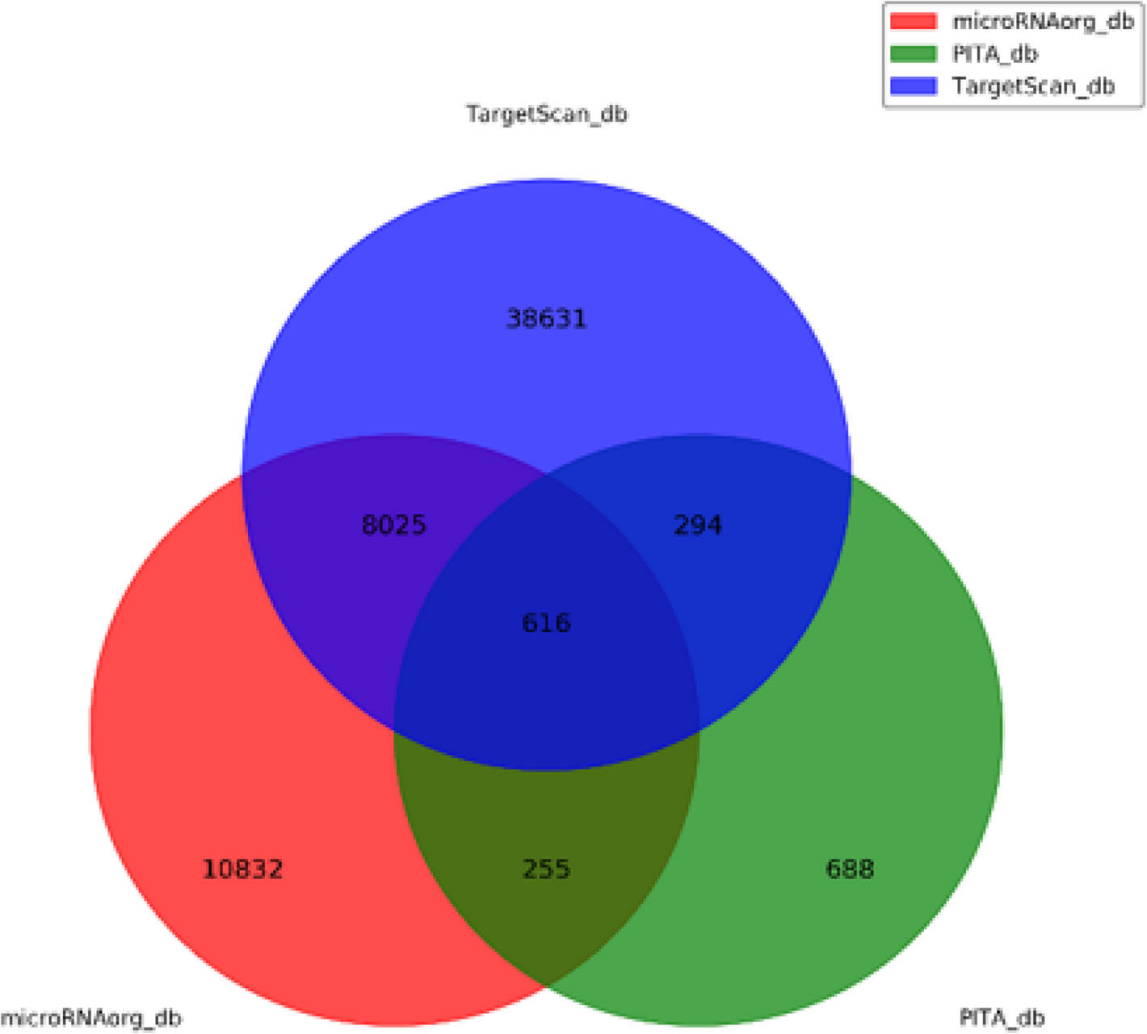
Target genes of differentially expressed miRNAs were the intersection predicted with 3 databases (Targetscan, microRNAorg, PITA)

**Fig. 7:**
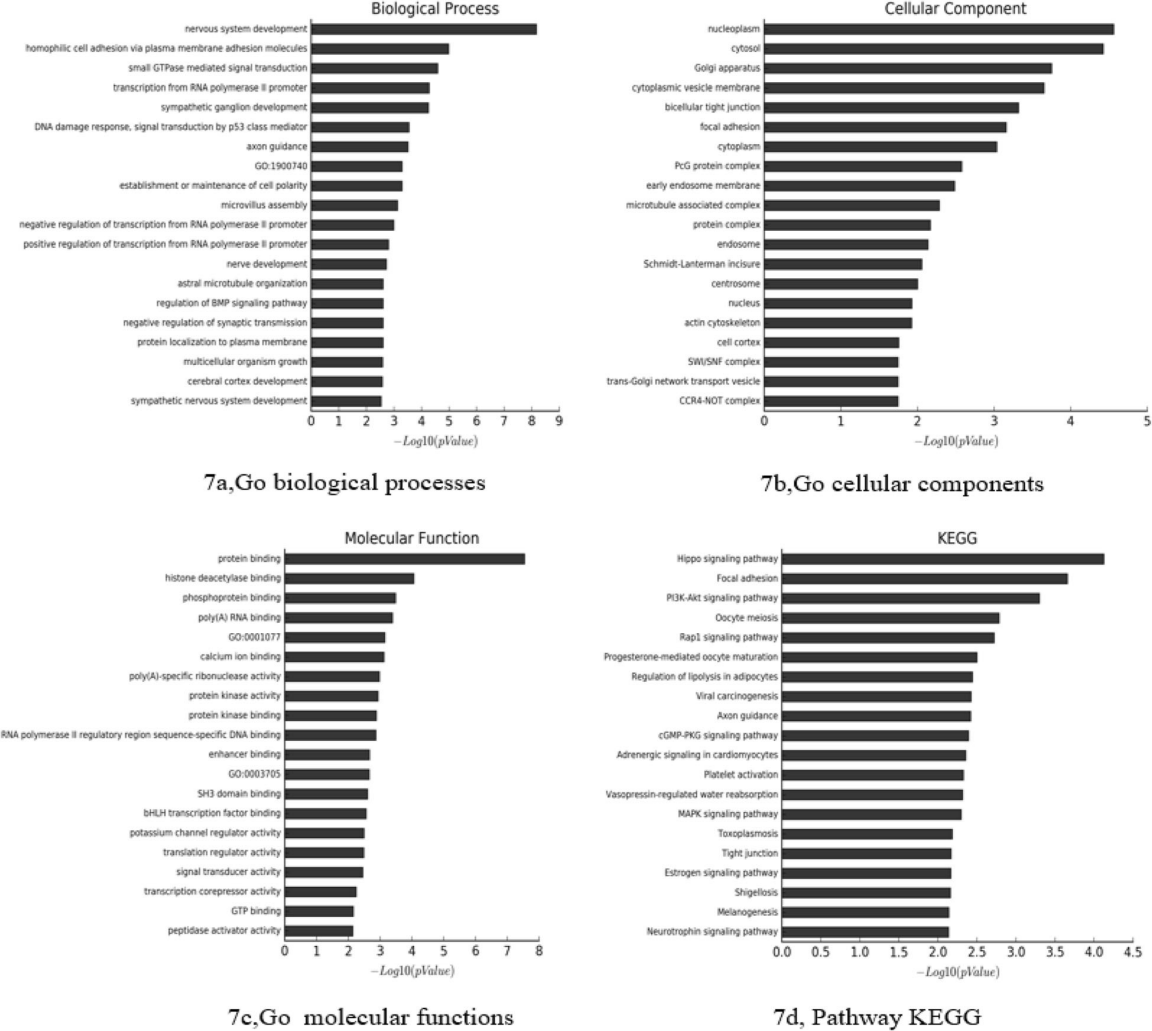
Bioinformation analysis of the differently expressed microRNAs and their predicted targets **a**: GO biological process; **b**: GO cellular component; **c**: GO molecular function; **d**: Pathway KEGG)

MiRNAs play a vital role in regulating life activities. Circulating miRNAs have been proved to be closely related to the development of many diseases, such as lung cancer, breast cancer, diabetes, metabolic syndrome, and heart failure, gradually becoming reliable biomarkers for various diseases [16, 18, 19]. There are many miRNAs that participate in the pathogenesis of atherosclerosis, mainly involved in lipid metabolism, inflammation, and endothelial dysfunction, thereby indicating that miRNAs are important factors in regulating the occurrence and development of atherosclerosis [20–22]. Circulating miRNAs were commonly considered to be derived from myocardial injury and atherosclerotic plaque rupture. Numerous studies have reported certain specific abnormal expression of circulating miRNAs of patients with ASCVD, suggesting that circulating miRNAs could be used as potential markers for the diagnosis of ASCVD [23, 24]. In this study, we used miRNA microarrays to analyze the differences of serum miRNA levels between the two groups (Additional file 1: Table S2 and Figure S2). We found that at least 12 miRNAs were upregulated in the LDL group, while 10 miRNAs were downregulated. Target genes of these differentially expressed miRNAs were the intersection predicted with 3 databases (Targetscan, microRNAorg, PITA). Using this method, a total of 616 target genes were found (Fig. 6). We used GO analysis to analyze all the target genes by DAVID, and we chose GOTERM_BP_FAT, GOTERM_CC_FAT, and GOTERM_MF_FAT. The analysis showed that the target genes were enriched in regulation of nervous system development, homophilic cell adhesion via plasma membrane adhesion molecules, small GTPase mediated signal transduction, mtranscription from RNA polymerase II promoter, sympathetic ganglion development and other biological processes in the GOTERM_BP_FAT (Fig. 7(a)), and were enriched in nucleoplasm, cytosol, golgi apparatus, cytoplasmic vesicle membrane, bicellular tight junction and other cellular components in the GOTERM_CC_FAT (Fig. 7(b)). In the GOTERM_MF_FAT, the target genes were enriched in protein binding, histone deacetylase binding, phosphoprotein binding, poly(A) RNA binding and other molecular functions (Fig. 7(c)). Finally, we performed the pathway analysis to analyze all the target genes by the KEGG. The results showed that in the classical pathway database KEGG, the target genes were enriched in hippo signaling pathway, focal adhesion, PI3K-Akt signaling pathway, oocyte meiosis, rap1 signaling pathway and other pathways (Fig. 7(d)). By analyzing Pathway analysis results, we found that among the top 20 signal pathways with the most target gene enrichment, 13 pathways contain the target gene of miRNA-933, including: CCDC97, BDNF, CAND1, DMRTA2, MAP4K4, PRKACB, MID1IP1, SLC16A2, ADCY9, FCHSD2, LRPAP1, etc. Pharmacogenomics studies have shown that the ADCY9 genotype determines the effects of CETP (cholesteryl ester transfer protein) inhibitor dalcetrapib on cardiovascular events and atherosclerosis [25], although the specific mechanism is not clear. BDNF was also reported play an important role in coronary atherosclerosis development [26]. However, whether miRNA-933 participates in the regulation of LDL-C is still not clear. Nevertheless, many studies mentioned that miRNA-933 could stably exist in the peripheral blood circulation [27, 28]. Previous studies on serum miRNA, such as acute myocardial infarction, coronary heart disease, and cerebral stroke, etc. [29–31], have proved that miRNA-933 could not only exist stably in the peripheral blood but also possessed significant differences when compared with control groups. However, few of these studies conducted further research on the correlation between circulating miRNA-933 and hyperlipidemia. Therefore we then conducted a preliminary exploration of the relationship between miRNA-933 and hyper-LDL-C and ASCVD.

Through miRNA microarrays, 22 miRNAs were found differentially expressed between the two groups. In order to verify the results, three miRNAs with relatively stable expression (i.e., miRNA-191-3p, miRNA-933, and miRNA-425-3p), as well as significant differences between the two groups, were selected for further PCR validation. The preliminary verification results of miR-191-3p, miR-933, and miR-425-3p were consistent with the miRNA microarray; all revealed a downregulation tendency. Further study of miRNA-933 in both groups verified the result, thereby suggesting that circulating miRNA-933 is probably related with a disorder of serum lipid metabolism (Additional file 1: Table S3). We then analyzed miRNA-933 expression between ASCVD and non-ASCVD group. The result showed miRNA-933 were a independent predictors associated with ASCVD by multivariate analysis. ROC curve analysis showed the area under the curve (AUC) was 0.739 (95% CI, 0.682–0.795; *P* = 0.00) and 0.703 (95% CI, 0.643–0.763; *P* = 0.00) in distinguishing the hyperlipidemia and the ASCVD patients, respectively. From the perspective of clinical application, We believe miRNA-933 could be helpful to identify patients with ASCVD at early stages. Additionally, as all the samples of this study were collected from clinical patients, we think these miRNAs may be more meaningful than the miRNAs found from animal models and therefore would be more helpful to reveal the potential mechanisms for lipid metabolism disorders. The limitation of this study include that the sample of the study was small and the results should be further confirmed in a large cohort patients.

## Conclusion

In summary, our study screened out several miRNAs (miRNA-191-3p, miRNA-933, and miRNA-425-3p etc.) with differential expression in the peripheral circulation of patients with lipid metabolism disorders (mainly LDL-C). Circulating miRNA-933 could be a feasible predictor for ASCVD at the early stage.

## Additional file

Additional file 1: **Table S1.** Logistic regression to predict ASCVD. **Table S2.** Basic information of RNA sample quality. **Table S3.** Logistic regression to predict hyper-LDL-C. **Figure S1.** Pearson correlation to analyzed the correlation between LDL-C and miR-933. **Figure S2.** The Electrophoregram of miRNA assessed for microarray. (DOCX 137 kb)

## Abbreviations

ADCY9: Adenylate cyclase 9
ALT: Alanine transaminase
AS: Atherosclerosis
ASCVD: Atherosclerotic cardiovascular disease
AST: Aspartate aminotransferase
BDNF: brain derived neurotrophic factor
BMI: Body mass index
CAND1: Cullin associated and neddylation dissociated 1
CCDC97: Coiled-coil domain containing 97
CVD: Cardiovascular disease
DMRTA2: DMRT like family A2
FCHSD2: FCH and double SH3 domains 2
HDL-C: High density lipoprotein cholesterol
LDL-C: Low-density lipoprotein cholesterol
LRPAP1: LDL receptor related protein associated protein 1
MAP4K4: Mitogen-activated protein kinase kinase kinase kinase 4
MID1IP1: MID1 interacting protein 1
PRKACB: Protein kinase cAMP-activated catalytic subunit beta
SD: Standard deviation
SLC16A2: Solute carrier family 16 member 2
TC: Total cholesterol
TG: Triglyceride
