# Supplementary material for: Several circulating miRNAs related to hyperlipidemia and atherosclerotic cardiovascular diseases
Source: Lipids Health Dis. 2019 Apr 22;18:104. doi: 10.1186/s12944-019-1046-z (PMC6477752; doi:10.1186/s12944-019-1046-z)
Supplement: Supplementary file 1 — Table S1. Logistic regression to predict ASCVD. Table S2. Basic information of RNA sample quality. Table S3. Logistic regression to predict hyper-LDL-C. Figure S1. Pearson correlation to analyzed the correlation between LDL-C and miR-933. Figure S2. The Electrophoregram of miRNA assessed for microarray. (DOCX 137 kb) [file 12944_2019_1046_MOESM1_ESM.docx]

Supplementary Material

Table 1 Logistic regression to predict ASCVD

|  | P value | OR | 95% CI | |
| --- | --- | --- | --- | --- |
| LDL | 0.000 | 4.393 | 2.088-9.243 | |
| Smoker | 0.298 | 1.579 | 0.204-1.627 | |
| TC | 0.003 | 5.613 | 2.862-11.006 | |
| HDL | 0.087 | 0.509 | 0.640-9.830 | |
| TG | 0.021 | 1.927 | 1.374-2.702 | |
| Serum miR-933 | | | | |
| First quartile(＜0.31) | 0.000 | 10.711 | 2.834-40.481 | |
| Second quartile (.31-0.64) | 0.018 | 5.255 | 0.875-12.111 | |
| Third quartile (0.64-1.89) | 0.077 | 3.276 | 1.352-20.595 | |
| Fourth quartile (＞1.89） |  |  |  |  |

Table 2


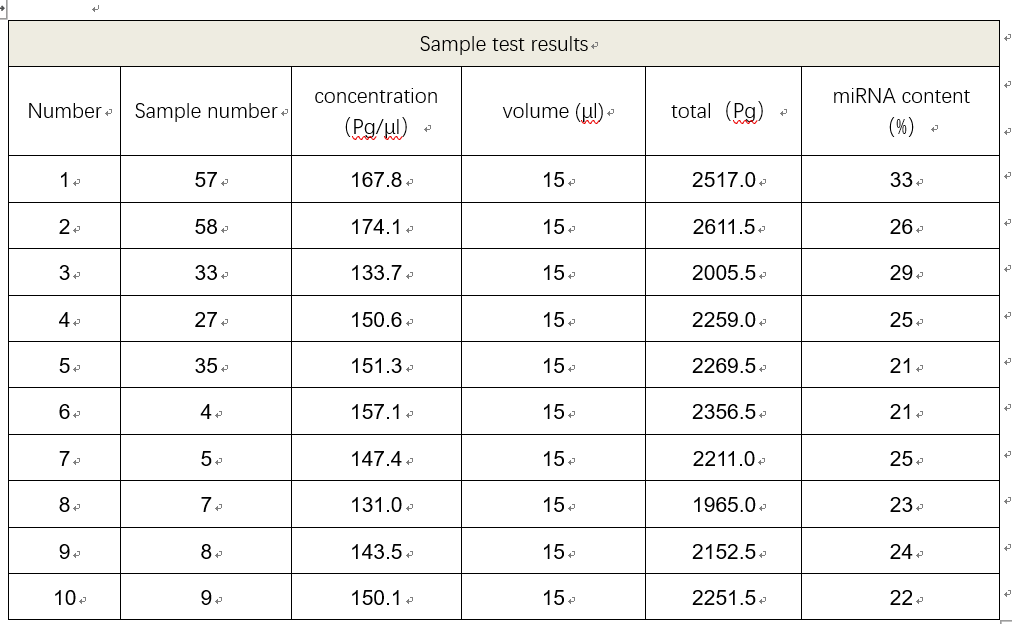


Table 3 Logistic regression to predict hyper-LDL-C

|  | P value | OR | 95% CI |
| --- | --- | --- | --- |
| miR-933 | 0.018 | 0.339 | 0.138-0.829 |
| TC | 0.000 | 23.464 | 10.650-51.659 |
| TG | 0.051 | 1.444 | 0.998-2.089 |
| HDL | 0.002 | 0.052 | 0.008-0.337 |
| UA | 0.252 | 0.997 | 0.991-1.002 |
| BUN | 0.269 | 0.852 | 0.641-1.132 |
| BMI | 0.140 | 1.133 | 0.960-1.337 |
| ASCVD | 0.041 | 1.744 | 0.675-4.503 |

Fig 1


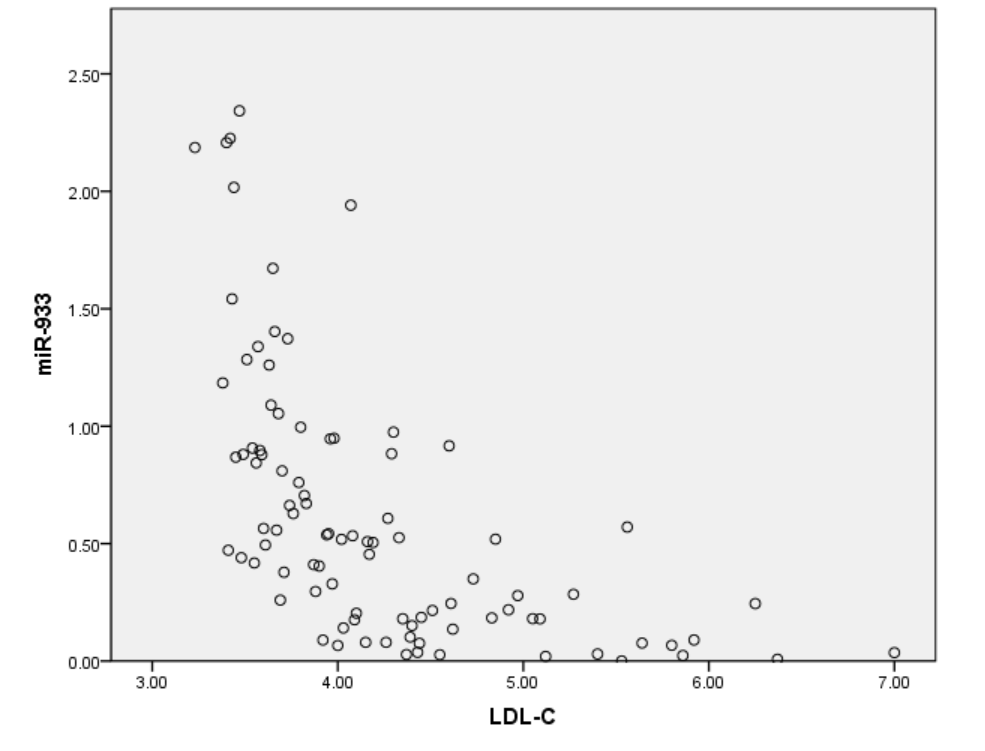


Fig.1. Pearson correlation to analyzed the correlation between LDL-C and miR-933.

Fig.2


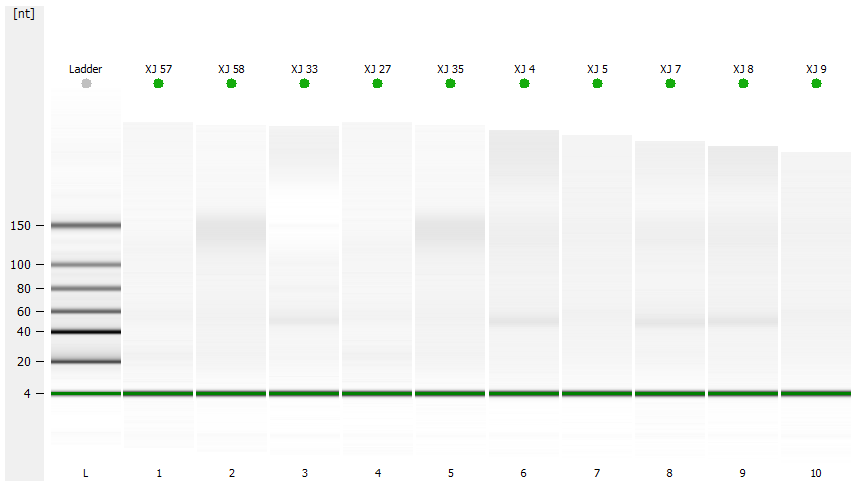


Fig.2. The Electrophoregram of miRNA assessed for microarray.
